# Parenting and family self-sufficiency services contribute to impacts of Early Head Start for children and families

**DOI:** 10.3389/fpsyg.2023.1302687

**Published:** 2023-12-14

**Authors:** Rachel Chazan-Cohen, Adam Von Ende, Caitlin Lombardi

**Affiliations:** ^1^Department of Human Development and Family Sciences, University of Connecticut, Storrs, CT, United States; ^2^Division of Developmental Medicine, Brazelton Touchpoints Center, Boston Children’s Hospital, Boston, MA, United States

**Keywords:** Early Head Start, parenting, family self-sufficiency, two-generation, resilience, infant and toddler

## Abstract

**Introduction:**

There is evidence that two-generation early childhood programs, those that strive to support not only child development, but also optimal parenting and family wellbeing, help to foster resilience for young children and their families in the face of adversity.

**Methods:**

Using data from a large experimental evaluation, the Early Head Start Research and Evaluation Project, this paper explores how parenting and family self-sufficiency services embedded in Early Head Start (EHS), a federally funded, nationally implemented two-generation early childhood program for low-income families lasting from pregnancy and until children are three, contribute to the impacts of the program for both the children and their families.

**Results:**

Parenting support in any modality (home visiting, case management or parent education) contributed to program impacts on important child and family outcomes, but not parent employment. Somewhat surprisingly, family receipt of employment services did not lead to any of the impacts of the program, while education and job training services did. When EHS parents received education or job training services, it led to impacts not only on mother employment, but also on other important family and child outcomes.

**Discussion:**

These findings validate and reinforce the two-generation approach of EHS, specifically supporting the focus on parenting and parent education and job training.

## 1 Introduction

Many early childhood programs strive to support not only child development, but also optimal parenting and family wellbeing (e.g., Chase-Lansdale and Brooks-Gunn, 2014; Haskins et al., 2014; Dropkin and Jauregui, 2015; Gruendel, 2015). While there is evidence that two-generation early childhood programs help to foster resilience for young children and their families in the face of adversity (Ludwig and Miller, 2007; Martin et al., 2008; Deming, 2009; Campbell et al., 2012; Love et al., 2013; Sabol and Chase, 2015), less is known about how specific parenting and family self-sufficiency services are part of the processes that lead to positive impacts. This paper explores how parenting and family self-sufficiency services embedded in Early Head Start, a federally funded, nationally implemented two-generation early childhood program for low-income families during pregnancy and until their children are three, contribute to the impacts of the program for both children and families. We first describe the EHS program and review the literature on the impacts of EHS and then summarize the literature on how early services aimed at parenting and family self-sufficiency have impacted children and families.

### 1.1 Early Head Start program and impacts

Early Head Start (EHS) was created with bipartisan support through the reauthorization of the Head Start Act in 1994. In 2021–2022, the federal EHS program served over 190,000 children, making it one of the largest programs serving low-income infants and toddlers in the US, although it still only serves approximately 10% of eligible children (National Head Start Association [NHSA], 2023). The primary emphasis of Early Head Start is on supporting child development with the ultimate goal of child competence in both the academic and social domains they need to succeed as they enter the formal school system (Administration for Children and Families [ACF], 2003). However, there is also an imperative to support parenting and family self-sufficiency as well, given that all EHS programs must follow the high standards for comprehensive two-generation services for families set by the Head Start Performance Standards (HSPPS; U.S. Department of Health and Human Services, 2016), the regulations that guide Head Start. Programs are assessed on whether they meet the HSPPS and can lose their federal grant funding if they are deemed to be not in compliance. While the HSPPS set high expectations for the quality, dosage, and breadth of services that families must receive, they do allow flexibility for programs to tailor services to meet the unique needs of families in their communities. Families can be enrolled in one of several program options, including home-based (one 90-min home visit a week along with regular group peer socialization activities), center-based child care (full day-full year group child care for at least 6 h a day along with at least 2 family support visits per year), family child care (similar to center-based option but child care is offered in a home or family-like setting), or a locally designed option. As of 2021–2022, approximately 63% of children were enrolled in the center-based option, 30% in the home-based, 4% in family child care, and 3% in another option (National Head Start Association [NHSA], 2023).

Whichever program option is applied, all EHS programs address the whole child and the whole family in the provision of services. A key underlying concept in EHS is that to optimally support the development of young children from low-income families it is necessary to not only provide the young children with high quality early care and education, but also to strengthen the family home environment; environments to which children will be exposed not only while they are participating in EHS, but also long afterward. EHS aims to address ongoing economic and psychosocial stressors in low-income families, helping families to change trajectories in lasting ways, and at the same time to support parents’ confidence and competence in the parenting role. Research on nationally representative samples of children enrolled in EHS has demonstrated that nearly all programs offer families comprehensive services aimed at increasing self-sufficiency and wellbeing, though the types of services vary (Vogel et al., 2015).

At the time that EHS was authorized, Congress called for a rigorous study of program impacts. A large national experimental impact study of EHS was conducted, the Early Head Start Research and Evaluation Project (EHSREP) with data collection points when children were 1, 2, 3, entering kindergarten, and in 5th grade. Findings are presented in many government reports and peer reviewed articles (Administration for Children and Families [ACF], 2002; Love et al., 2005, 2013; Vogel et al., 2011). In sum, when children were in the program, at ages 2 and 3 there was a pattern of all positive, but modest in magnitude, impacts across an array of child and family outcomes. Impacts for children included impacts on all domains of child development assessed (health, social-emotional, cognitive, language), including fewer visits to the emergency room due to accident and injury. EHS parents, both mothers and fathers, were more supportive and less negative in play interactions with their children and provided home environments that were more supportive of learning and development. EHS also reduced punitive parenting, including spanking, by both mothers and fathers. Furthermore, mothers who had been in EHS were more likely to be employed or in education or job training (Vogel et al., 2013). Not surprisingly, EHS families also reported receiving a greater array of services than the families in the control group (Administration for Children and Families [ACF], 2002). See Supplementary Table 1 for a list of services that EHS impacted. Two years after the program ended, positive impacts remained in the areas of children’s social-emotional outcomes, parenting, and parent wellbeing (Chazan-Cohen et al., 2013a; Love et al., 2013). In 5th grade, eight years after the end of the EHS program, the only remaining overall impact among the 54% of the original sample that was successfully located was a trend for more positive child social-emotional wellbeing, although patterns of impacts varied for different subgroups of families, with some continuing to benefit from the program (Vogel et al., 2011). A longer-term follow up looking at children’s involvement in the child welfare system through their first 15 years of life showed that EHS has an impact on the likelihood of child welfare system involvement that is driven by earlier impacts on parenting behaviors, family wellbeing, and child developmental status (Green et al., 2020).

### 1.2 Two-generation early childhood programs—whole child/whole family

Two-generation early childhood programs have based their emphasis on supporting parenting on the long history of developmental science that includes both theory and empirical evidence linking parenting behaviors with child outcomes (Halle et al., 2011; National Academies of Sciences, Engineering, and Medicine, 2016). In fact, one of the founders of Head Start was Urie Bronfenbrenner, whose bio-ecological approach to child development put forth the idea that to have lasting effects for children, interventions must strive for consistency across community, home and educational setting (Zigler and Muenchow, 1992). His voice was instrumental in how Head Start programs approached family engagement.

There is evidence that current-day two-generation programs are effective in influencing both child and family outcomes. In addition to the EHS findings detailed above, there is a growing literature on the effectiveness of home visiting programs to influence both parenting and child outcomes, although patterns are stronger for parent outcomes (Howard and Brooks-Gunn, 2009). Findings from the many studies of home visiting have found different patterns of impacts for different home visiting models as well as varying impacts depending on aspects of the community in which the intervention is conducted (Sama-Miller et al., 2016). However, broadly speaking, home visiting has been found to have the most consistent positive impacts on positive parenting practices, including reductions in child maltreatment (Howard and Brooks-Gunn, 2009; National Academies of Sciences, Engineering, and Medicine, 2016; Sama-Miller et al., 2016). Fewer studies have included measures of family self-sufficiency outcomes, but some have found impacts on larger spacing between childbearing (Olds et al., 2004) and increased education and job training (Jones Harden et al., 2012; Chazan-Cohen et al., 2013b). Many home visiting models emerged from the health sciences and healthcare settings and have documented improvements in access to healthcare and child health, including fewer visits to the emergency room due to accidents and injuries (Koniak-Griffin et al., 2003; Dodge et al., 2013). Some home visiting models, including the EHS home-based option, have documented positive impacts on child development outcomes and school readiness (Lowell et al., 2011; Jones Harden et al., 2012; Raikes et al., 2023). Importantly, these positive impacts for children tended to emerge in programs that focused on child development (Jones Harden et al., 2012; Raikes et al., 2023) and where home visit time is spent specifically on child development (Raikes et al., 2006; Roggman et al., 2016).

Fewer studies have been done of how to address the whole family, including supporting parenting and family self-sufficiency, in early childhood programs that are primarily group care based, either center based child care or family child care. Promising avenues that are often overlapping, include parent education, case management, parenting groups and parent coaching (Jeong et al., 2021).

Little research has addressed the effectiveness of the addition of parent education, both in group parenting classes as well as in more individualized and informal interactions, to early childhood group care settings or as components of comprehensive programs like EHS. Much of the literature about these programs summarizes how to engage families—why parents join groups and barriers to participation—rather than impacts of the programs (National Academies of Sciences, Engineering, and Medicine, 2016). There are a few instances of manualized parenting support programs being embedded successfully in Head Start. One parenting program that included parent groups, the Incredible Years, was implemented in conjunction with preschool Head Start and was found to be effective in reducing negative parenting and child conduct problems (Webster-Stratton and Reid, 2007). The new Incredible Years Parents and Babies program has not yet been studied in the context of EHS, although preliminary evaluation studies have shown this group-based parenting intervention to be promising in impacting parenting behaviors (Pontoppidan et al., 2016; Hutchings et al., 2017). Another more clinically intensive, although brief, parenting program, consisting of 10 home visits, the Attachment and Biobehavioral Catch-up, has been successfully embedded in a home-based EHS program. A randomized controlled study found impacts on a variety of maternal behaviors assessed in interaction with her baby, as well as child cortisol levels (Berlin et al., 2018, 2019).

Ample evidence has also documented the important role of supporting family self-sufficiency, specifically through supporting parental employment and education and job training on both parent and child outcomes. Broadly, decades of research have documented the positive impacts that parental education, family income, and parental employment have on parental wellbeing and children’s development (Duncan et al., 2014; Heinrich, 2014; Davis-Kean et al., 2021). There are mixed results from research on two-generation interventions that included education and job-related support for mothers as well as child care to support mothers’ employment, without a focus on the quality of that care. Conversely, two-generation interventions that include support for family self-sufficiency as well as high quality child care have had more consistently positive results for both parental outcomes as well as children’s development (for a review, see Chase-Lansdale and Brooks-Gunn, 2014; National Academies of Sciences, Engineering, and Medicine, 2016). These programs include the Child-Parent Center Program in Chicago, CareerAdvance Community Action Project of Tulsa, Oklahoma, the Annie E. Casey Foundation Atlanta Partnership, and the Housing Opportunity and Services projects.

### 1.3 The current study

In sum, there is empirical support for the importance of the focus on both parenting and family self-sufficiency within EHS. Not only do EHS programs support programs in these areas, but they are also fertile settings to embed specialized interventions. Surprisingly, very little research has been done to show how these aspects of the two-generation services provided by EHS are associated with later impacts for both children and families. This paper will show how key EHS services mediate the impacts of the program. Specifically, our research questions are:

(1) How do impacts of EHS on receipt of parenting support, through case management, home visiting and parent group education experiences at age 2, lead to impacts on children and families at age 3?

(2) How do impacts of EHS on receipt of employment and education support at age 2 lead to impacts for children and families at age 3?

## 2 Materials and methods

### 2.1 Sample

The Early Head Start Research and Evaluation project (EHSREP) is a randomized evaluation of EHS conducted in 17 communities across the US. It included 3,001 families who had children under the age of 1 at the time of enrollment (average age was approximately 3 months and 1/4 of the sample enrolled during pregnancy). The project includes a myriad of child and family measures collected when children were 1, 2, and 3 years of age, and at pre-kindergarten and fifth grade. In this article, we report on services that families report receiving at approximately age 2 and child and family outcomes at age 3.

The sample for the current study includes 2,977 EHS study participants (1,503 randomly assigned to the EHS program, and 1,474 controls) originally included in the randomized study. Sample demographic characteristics are shown in Table 1. Slightly more than half of the children in the sample (51%) were male. Families were racially and ethnically diverse, with 37% self-identifying as White, 35% as Black and 24% as Hispanic. Fifty-three percent of mothers were not working or in school at the time of randomization, 46% did not have a high school degree or GED, 38% were teens when they became mothers, 28% received governmental assistance (TANF or AFDC), and 7.5% reported being homeless at some point in their lives.

**TABLE 1:** Demographic information at randomization (*n* = 2,977).

Characteristic	*N* (%)
EHS program	1,503 (50.5%)
Focus child is male	1,502 (50.9%)
**Ethnicity**	
White	1,086 (37.1%)
Black	1,014 (34.7%)
Hispanic	692 (23.7%)
Other	133 (4.5%)
**Demographic risk indicator**	
Ever homeless	224 (7.5%)
Not working or in school	1,581 (53.1%)
Teen mother	1,140 (38.3%)
Receives government assistance	837 (28.1%)
No high school diploma or equivalent	1,367 (45.9%)

### 2.2 Measures

#### 2.2.1 Family use of services

At regular intervals post randomization, 6-months, 15-months and 26-months, all families were interviewed about their use of services. In this paper we utilize family report of (1) home visiting at least once per month, (2) home visiting that addressed child development and parenting (3) case management at least once per month, (4) case management related to parenting, (5) parenting education, (6) parenting groups, (7) education and job training services, and (8) employment services. All questions were yes/no, whether they had received the service or not. We calculated whether the family had ever reported receiving these services at any point up until the 26-month assessment. These were all services where previous research has shown that EHS had an impact, see Supplementary Table 1 for rates of service use for the EHS and control group.

#### 2.2.2 Child outcome measures

Bayley Scales of Infant and Toddler Development, Mental Development Index (MDI) (Bayley, 1993). The Bayley MDI is a cognitive assessment used to identify developmental delays in young children. The Bayley MDI was conducted at 36 months of age by trained assessors. In the norming sample, MDI internal reliability was 0.88, test-retest reliability ranged from 0.77 to 0.91, and the MDI was correlated with other tests of cognitive functioning, including the McCarthy Scales of Children’s Abilities (0.79) and the Wechsler Preschool and Primary Scale of Intelligence-Revised (0.73). Total raw scores were calculated and then transformed into standard scores.

Peabody Picture Vocabulary Test, Third Edition (PPVT III) (Dunn and Dunn, 1997). The PPVT-III is an assessment of vocabulary comprehension in standard English. The child is presented with four pictures and is asked to point to the picture that matches the word spoken by the interviewer. The PPVT III has good internal consistency reliability (Cronbach’s alpha = 0.92–0.98) and correlates highly (0.8–0.9) with intelligence tests. Total raw scores were calculated and then transformed into standard scores.

The 3-bag Assessment, Child Engagement with Parent During Play (Owen et al., 2002). During the research home visit at age 36 months, parents were given three bags of interesting toys and asked to play in sequence. Interactions were videotaped and child and parent behaviors were coded by a national coding team to capture a number of dimensions on 7-point scales. Interrater reliability was high, ranging from 87–96 percent across dimensions (Fuligni and Brooks-Gunn, 2013). We used the Child Engagement with Parent scale which rated child’s bids for interaction with the mother as well as behaviors expressing positive regard toward the mother.

#### 2.2.3 Family outcome measures

Home observation for measurement of the environment (HOME) (Bradley and Caldwell, 1988). The HOME is one of the most widely used measures of parent support for learning and development and includes both observational and interview items. The HOME was collected at 36 months of age during a research data collection home visit. Cronbach’s alpha was 0.80 for this sample (Administration for Children and Families [ACF], 2002).

Parent report of spanking. Parent interviews at 36 months included a single question assessing whether parents spanked the child within the past week.

Parent report of employment. Parents interviews at 36-month is included a single question asking whether parents were employed.

### 2.3 Analytic plan

We used structural equation modeling (SEM) to estimate indirect effects of the EHS intervention on age 3 child and family outcomes through EHS services-related variables collected at age 2. Services-related variables impacted by the EHS intervention in earlier reports were selected as mediators. To combine information and reduce measurement error in observed mediation variables, we created latent constructs for three of the service-related domains (home visiting, case management, and parenting education services). In particular, the latent construct for “home visiting” consisted of: at least one home visit per month, receipt of child development services, and receipt of parenting information in home visiting; “case management” consisted of case management at least once per month and discussion of parenting with the case manager; and “parenting education” consisted of participation in any group parenting activity and receipt of parenting education services.

For each of the six mediators, we constructed two distinct models: one including the three child outcomes and another including the three family outcomes, yielding a total of 12 models. Indirect effects were tested using standard errors derived from bootstrapping. Model fit was evaluated using multiple fit indices, including the chi-squared difference between observed and expected covariance matrices. While a non-significant chi-squared test is often indicative of good fit, this statistic is sensitive to sample size and is often significant in large samples even when model fit is acceptable. Other fit indices included the root mean square error of approximation (RMSEA), where values less than 0.05 are favorable, as well as the comparative fit index (CFI) and Tucker-Lewis index (TLI), where values exceeding 0.95 are desirable. Furthermore, we assessed the standardized root mean square residual (SRMR), with values below 0.08 indicating a favorable model fit.

Missing data was handled using full information maximum likelihood (FIML). Baseline covariates for mediator and outcomes models included child age, sex, race (Black, Hispanic, White, and other), and demographic risk index (a sum of teen motherhood, no maternal high school education, receipt of government assistance, ever homeless, and currently unemployed).

#### 2.3.1 Supplemental models

To assess whether there are meaningful differences between EHS and control participants who contribute information to the analyses, we compared EHS and control participants who had non-missing data for at least one mediation and at least one outcome on 19 characteristics. These characteristics were used as adjustment variables in the original EHSREP impact study. Of the 19, only self-report of “inadequate food” at recruitment differed between the two groups (*P* = 0.01; Supplementary Table 4). After re-running the models while further adjusting for “inadequate food,” the results were unchanged. To assess the robustness of the results to the treatment of missing data, we conducted a sensitivity analysis where we used listwise deletion of missing data instead of FIML. See Supplementary Table 5 for results of these supplemental models.

## 3 Results

Descriptive statistics for all mediators and outcomes are displayed in Supplementary Table 1, and all direct effect estimates (i.e., the effects of the EHS program on the mediator and the effects of the mediator on the child and parenting outcome) are displayed in Supplementary Table 2.

### 3.1 Child outcomes

**FIGURE 1:**
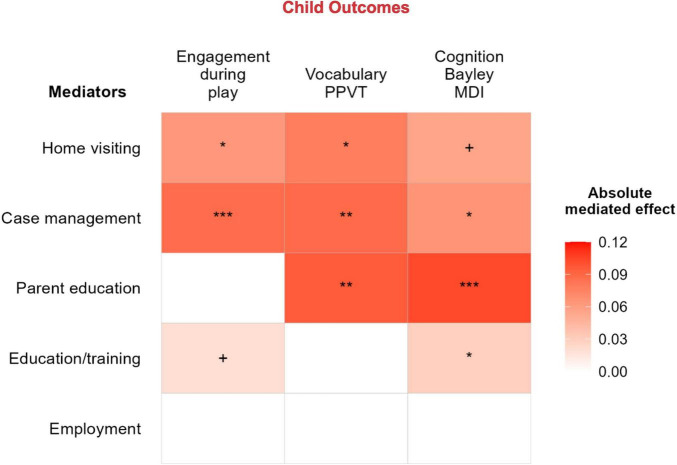
Estimates of the indirect effects of parenting and self-sufficiency services on child outcomes. ***p < 0.001, **p < 0.01, and *p < 0.05.

As shown in Table 2 and Figure 1, the effect of EHS on child engagement during play was mediated by home visiting (Estimate = 0.063, SE = 0.029, *p* = 0.029), case management (Estimate = 0.087, SE = 0.023, *p* < 0.001), education/training services (Estimate = 0.020, SE = 0.011, *p* = 0.058; trend), but not group parenting or employment services. The effect of EHS on PPVT scores was mediated by home visiting (Estimate = 0.078, SE = 0.033, *p* = 0.020), case management (Estimate = 0.088, SE = 0.027, *p* = 0.001), and group parenting (Estimate = 0.091, SE = 0.064, *p* = 0.003), but not education/training or employment services. The effect of EHS on MDI scores was mediated by home visiting (Estimate = 0.056, SE = 0.032, *p* = 0.082; trend), case management (Estimate = 0.065, SE = 0.026, *p* = 0.012), group parenting (Estimate = 0.103, SE = 0.062, *p* < 0.001) and education/training services (Estimate = 0.030, SE = 0.012, *p* = 0.012), but not employment services.

**TABLE 2:** Unstandardized estimates of the indirect effects of parenting and self-sufficiency services on child and family outcomes.

Model	Mediator	Outcome	Estimate	SE	*P*-value
1	Home visiting	Child engagement	0.063	0.029	0.029
1	Home visiting	PPVT	0.078	0.033	0.020
1	Home visiting	MDI	0.056	0.032	0.082
2	Case management	Child engagement	0.087	0.023	< 0.001
2	Case management	PPVT	0.088	0.027	0.001
2	Case management	MDI	0.065	0.026	0.012
3	Group parenting	Child engagement	0.072	0.052	0.166
3	Group parenting	PPVT	0.091	0.064	0.003
3	Group parenting	MDI	0.103	0.062	< 0.001
4	Education/training services	Child engagement	0.020	0.011	0.058
4	Education/training services	PPVT	0.014	0.012	0.232
4	Education/training services	MDI	0.030	0.012	0.012
5	Employment services	Child engagement	−0.001	0.010	0.939
5	Employment services	PPVT	0.000	0.011	0.965
5	Employment services	MDI	0.006	0.011	0.580
6	Home visiting	Home environment	0.072	0.019	< 0.001
6	Home visiting	Spanking	−0.036	0.014	0.013
6	Home visiting	Not working	0.012	0.014	0.386
7	Case management	Home environment	0.055	0.017	0.001
7	Case management	Spanking	−0.040	0.012	< 0.001
7	Case management	Not working	0.001	0.011	0.934
8	Group parenting	Home environment	0.103	0.041	< 0.001
8	Group parenting	Spanking	−0.012	0.027	0.663
8	Group parenting	Not working	−0.018	0.025	0.468
9	Education/training services	Home environment	0.028	0.007	< 0.001
9	Education/training services	Spanking	−0.012	0.005	0.028
9	Education/training services	Not working	−0.040	0.006	< 0.001
10	Employment services	Home environment	0.003	0.006	0.664
10	Employment services	Spanking	−0.004	0.005	0.382
10	Employment services	Not working	−0.005	0.005	0.245

### 3.2 Family outcomes

**FIGURE 2:**
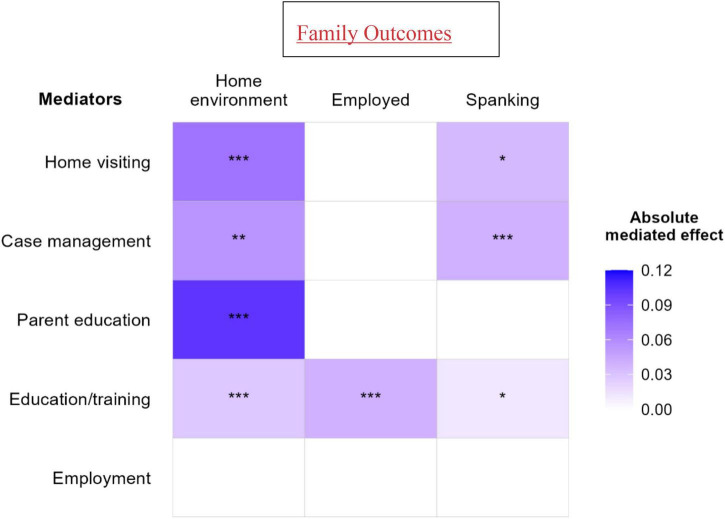
Estimates of the indirect effects of parenting and self-sufficiency services on family outcomes. ***p < 0.001, **p < 0.01, *p < 0.05.

As shown in Table 2 and Figure 2, the effect of EHS on the home environment was mediated by home visiting (Estimate = 0.072, SE = 0.019, *p* < 0.001), case management (Estimate = 0.055, SE = 0.017, *p* = 0.001), group parenting (Estimate = 0.103, SE = 0.041, *p* < 0.001), education/training services (Estimate = 0.028, SE = 0.007, *p* < 0.001), but not employment services. The effect of EHS on spanking was mediated by home visiting (Estimate = −0.036, SE = 0.014, *p* = 0.013), case management (Estimate = −0.040, SE = 0.012, *p* < 0.001), education/training services (Estimate = −0.012, SE = 0.005, *p* = 0.028), but not group parenting or employment services. The effect of EHS on employment status was mediated by education/training services (Estimate = −0.040, SE = 0.006, *p* < 0.001) but not employment services or any other services-related mediator.

### 3.3 Model fit

Model fit was excellent across all models. Across all 10 models, the mean RMSEA value was 0.017 (mean upper bound = 0.031), the mean CFI was 0.992, the mean TLI was 0.969, and the mean SRMR was 0.010. The model chi square was significant in 4 of the 10 models, though this is unsurprising given the large sample size.

## 4 Discussion

The goal of this study was to investigate how parenting and family self-sufficiency services within EHS promote resilience and support families and children’s development. We examined how impacts of EHS on receipt of parenting support and employment and education support at age 2 lead to impacts on children and families at age 3. Findings validate and reinforce the two-generation approach of EHS and the regulations (Head Start Program Performance Standards) that guide EHS programs, specifically supporting the focus on parenting and parent education and job training.

Knowledge of child development and parenting support provided through weekly home visits or less frequent case management led to program impacts on child engagement of their parent during play, as well as impacts on children’s vocabulary and cognitive development. Parent education services led to impacts on vocabulary and cognitive development, and in fact was the largest mediator of children’s cognitive development, but these services did not lead to impacts on children’s behavior with the parent in play interactions. It may be that individualized services like home visiting or case management are more likely to focus on parent-child interactions while group-based parent education may focus more on how to provide experiences and environments that stimulate language and cognition. Likewise, all forms of parenting support resulted in impacts on the warmth and stimulation provided in the home environment, although mediation was particularly strong for parenting education. Parenting support in home visits and case management contributed to the impacts on reduced punitive parenting, but group parenting education did not. Again, it is likely that discipline techniques were more likely to be addressed in individual home visiting or case management experiences. Parenting services did not contribute to the impact of the program on mothers being employed at the end of the program. In sum, providing parenting support in any modality contributed to program impacts on important child and parenting outcomes, but not parent employment.

Somewhat surprisingly, family receipt of employment services did not lead to any of the impacts of the program, even parent employment, while education and job training services did. When EHS parents received education or job training services, it led to impacts not only on mother employment, but also on other important family outcomes, both providing a warm and stimulating home environment and the child experiencing less spanking. To a lesser degree, education and job training contributed to impacts of the program on child engagement of the parent during play (a statistical trend) and on children’s cognitive development. It may be that helping the parent attain job skills or educational credentials helped parents not only get a job, but get a better job, that in turn resulted in more positive outcomes for children and their families. This coincides with existing research and theory finding that better quality employment is linked with enhanced child and family outcomes whereas low quality employment can be harmful, exerting stress without corresponding economic benefits (Menaghan and Parcel, 1995; Raver, 2003; Lombardi, 2021).

Our findings confirm the importance of the two-generation approach in early childhood education programs and contribute to the literature on the importance of parenting focused interventions for families with the youngest children (National Academies of Sciences, Engineering, and Medicine, 2016; Jeong et al., 2021). There is a growing literature on the effectiveness of interventions that coach parents, often using videotaped interactions. Positive impacts have been found for video-based parent coaching on parenting behaviors (Spieker et al., 2012), child social emotional outcomes (Weisleder et al., 2016; Mendelsohn et al., 2018), and language development (Ramırez et al., 2019). During the COVID pandemic some coaching programs transitioned to a virtual format and a recent systematic review conducted by van Ijzendoorn et al. (2023) found that this is an effective format. This flexibility in format may make this parenting coaching even more appealing to parents and to early childhood programs.

There is also some evidence that relationship-based parent groups can be an effective way to engage parents of very young children and result in more positive parenting as well as reduced stress and depression (Constantino et al., 2001; Pontoppidan et al., 2016; Hutchings et al., 2017; Cooke et al., 2023). Surprisingly, these programs had good attendance, despite the fact that families were adjusting to having a young child in the home. This suggests that a focus on early parenting is one way to engage families in parenting education in group settings, although the peer support aspect may also motivate parents to attend and may contribute to the positive impacts.

As noted in the introduction, Head Start programs are designed to be two-generation programs and this makes them an ideal setting in which to embed parenting and employment interventions. The effective parent coaching and parenting group intervention described above can be implemented and tested within EHS programs. It may be that for some families a group approach will be more optimal but other may prefer an individualized approach. Two-generation programs can also create partnerships with local businesses or institutions of higher education to offer training and career planning for parents. Staff and families in EHS are primed to do this work, and while the program already addresses parenting and self-sufficiency goals of families, these more targeted interventions can strengthen program effects.

### 4.1 Limitations

This was a secondary analysis of the existing EHSREP data. There are inherent limitations to secondary data analysis, including the historical context and the measures included. This study began in the late 1990’s and did not assess the breadth of comprehensive services that newer descriptive studies of EHS include (Vogel et al., 2011). It is likely that since the time of this study, families’ needs as well as the profile of available community services have changed. While current studies of EHS are descriptive and do not include a control group, these basic questions should be explored in more recent studies of EHS.

There are other statistical limitations to this work as well. There was a non-negligible amount of missing data for the variables under study; for example, about 30% of participants were missing data on mediators at age 2. However, these patterns were consistent across treatment and control groups, and we used statistical methods to include all participants in the analysis, which should provide unbiased and consistent results under the principle of intention-to-treat. The analyses include multiple mediators and outcomes, leading to numerous statistical tests, which could increase the likelihood of Type 1 errors. However, given we chose mediators for which we have strong *a priori* hypotheses regarding the nature and directionality of effects, we opted not to adjust for multiple comparisons, which is likely to be overly conservative and obscure meaningful relationships.

### 4.2 Next steps

Many if not all two-generation early childhood programs include aspects of parenting and family self-sufficiency support, either in group (parenting groups or socializations) or individual settings (case management and home visiting) and to varying degrees of intensity. While this study shows how important these aspects of services are, more qualitative research is needed to examine the specific ingredients that make these services important. For instance, does it matter who provides the parent support? Is parenting support best provided by a staff member who also works with children, or a staff member specifically trained to work with parents? What about the coordination between different staff members? We know that parents often do not avail themselves of parenting groups that are part of most two-generation models, what can be done to increase participation? Despite recent calls for more of a focus on fathers (Cabrera, 2020), most research focuses on how to engage fathers in interventions (Tully et al., 2017; McKee et al., 2021), rather than on the effectiveness engaging fathers in these efforts (Mihelic et al., 2018). We also need more recent datasets to explore how EHS programs are addressing parenting behaviors and family self-sufficiency in our current moment in history. More research is needed on how these services can be augmented and strengthened using targeted and intensive manualized approaches. Finally, research is needed to see if these services can be embedded in community early care and education settings as well as existing Early Head Start programs.

## 5 Implications and conclusion

We have long known that providing two-generation comprehensive services to families with young children can change the trajectories for both children and family wellbeing. The findings from this study help to articulate the contributions of services aimed at supporting parenting and family self-sufficiency. The findings support providing parenting support in whatever modality works for families, be it in home visiting, case management, or parenting education which is often provided in group settings. Furthermore, the focus on parenting education and training was more effective than focusing on parenting employment. These findings imply that not only should programs continue to provide these services but should also look to improve upon current approaches, perhaps by adding proven coaching and group parenting interventions for families who are interested. Research is needed to explore how these whole family services can be embedded in community early care and education settings as well as existing Early Head Start programs.

## Data availability statement

Publicly available datasets were analyzed in this study. The datasets analyzed for this study can be found at the Murray Research Archives at Harvard University https://dataverse.harvard.edu/dataset.xhtml?persistentId=doi:10.7910/DVN/TH7GEB.

## Author contributions

RC-C: Conceptualization, Funding acquisition, Supervision, Writing – original draft. AV: Formal analysis, Visualization, Writing – review & editing. CL: Methodology, Writing – review & editing.
