# Supplementary material for: Parenting and family self-sufficiency services contribute to impacts of Early Head Start for children and families
Source: Front Psychol. 2023 Dec 14;14:1302687. doi: 10.3389/fpsyg.2023.1302687 (PMC10752921; doi:10.3389/fpsyg.2023.1302687)
Supplement: Supplementary file 1 [file Data_Sheet_1.docx]

**Supplemental information**

Supplementary Table 1. Descriptive statistics for study variables, by EHS status

| **Variable** | **Controls** | **EHS** |
| --- | --- | --- |
| **Mediators** |  |  |
| Receipt of employment services | 191 (19.0%) | 399 (37.3%) |
| Receipt of education/training services | 325 (32.1%) | 575 (53.4%) |
| Home visits > 1 times per month | 49 (4.9%) | 494 (47.5%) |
| Receipt of parenting info in home visiting | 81 (8.0%) | 605 (56.3%) |
| Receipt of child development services in home visiting | 93 (9.2%) | 627 (58.4%) |
| Case management > 1 times per month | 112 (11.2%) | 504 (47.5%) |
| Discussed parenting with case manager | 180 (17.9%) | 572 (53.3%) |
| Any group parenting activity | 172 (17.0%) | 476 (44.4%) |
| Receipt of parenting education services | 620 (64.7%) | 1,007 (94.2%) |
| **Outcomes** |  |  |
| Child Engagement during play | 4.64 (1.04) | 4.78 (0.98) |
| Child Attention during play | 4.83 (0.98) | 4.95 (0.92) |
| PPVT | -0.04 (1.05) | 0.04 (0.95) |
| Bailey MDI | -0.05 (1.02) | 0.04 (0.98) |
| Home total score | 26.96 (4.90) | 27.48 (4.67) |
| Spanking | 517 (53.6%) | 502 (47.1%) |
| Parent supportiveness during | 3.87 (0.92) | 3.97 (0.93) |
| Not employed or in school | 302 (33.0%) | 302 (30.2%) |

Supplementary Table 2. Direct effects of EHS (‘X’) on age 2 mediators (‘M’), and age 2 mediators (‘M’) on age 3 child and parent outcomes (‘Y’)

| **Model** | **Path** | **Predictor** | **Outcome** | **Estimate** | **SE** | **P-value** |
| --- | --- | --- | --- | --- | --- | --- |
| Child outcomes at age 3 | | | | | | |
| 1 | X -> M | Program | Home visiting | 0.407 | 0.016 | <.001 |
| 1 | M -> Y | Home visiting | Child engagement | 0.154 | 0.071 | 0.029 |
| 1 | M -> Y | Home visiting | PPVT | 0.190 | 0.082 | 0.020 |
| 1 | M -> Y | Home visiting | MDI | 0.138 | 0.079 | 0.081 |
| 2 | X -> M | Program | Case management | 0.363 | 0.019 | <.001 |
| 2 | M -> Y | Case management | Child engagement | 0.241 | 0.064 | <.001 |
| 2 | M -> Y | Case management | PPVT | 0.243 | 0.074 | 0.001 |
| 2 | M -> Y | Case management | MDI | 0.180 | 0.071 | 0.012 |
| 3 | X -> M | Program | Parenting | 0.281 | 0.019 | <.001 |
| 3 | M -> Y | Group parenting | Child engagement | 0.258 | 0.186 | 0.166 |
| 3 | M -> Y | Group parenting | PPVT | 0.680 | 0.223 | 0.002 |
| 3 | M -> Y | Group parenting | MDI | 0.732 | 0.215 | <.001 |
| 4 | X -> M | Program | Education/training services | 0.215 | 0.021 | <.001 |
| 4 | M -> Y | Education/training services | Child engagement | 0.094 | 0.049 | 0.053 |
| 4 | M -> Y | Education/training services | PPVT | 0.066 | 0.055 | 0.229 |
| 4 | M -> Y | Education/training services | MDI | 0.139 | 0.054 | 0.010 |
| Parenting outcomes at age 3 | | | | | | |
| 5 | X -> M | Program | Employment services | 0.184 | 0.019 | <.001 |
| 5 | M -> Y | Employment services | Child engagement | -0.004 | 0.053 | 0.939 |
| 5 | M -> Y | Employment services | PPVT | 0.003 | 0.060 | 0.965 |
| 5 | M -> Y | Employment services | MDI | 0.033 | 0.059 | 0.579 |
| 6 | X -> M | Program | Home visiting | 0.407 | 0.016 | <.001 |
| 6 | M -> Y | Home visiting | Home environment | 0.176 | 0.047 | <.001 |
| 6 | M -> Y | Home visiting | Spanking | -0.089 | 0.035 | 0.012 |
| 6 | M -> Y | Home visiting | Not working | 0.029 | 0.033 | 0.386 |
| 7 | X -> M | Program | Case management | 0.363 | 0.019 | <.001 |
| 7 | M -> Y | Case management | Home environment | 0.152 | 0.047 | 0.001 |
| 7 | M -> Y | Case management | Spanking | -0.11 | 0.032 | <.001 |
| 7 | M -> Y | Case management | Not working | 0.003 | 0.030 | 0.934 |
| 8 | X -> M | Program | Group parenting | 0.283 | 0.019 | <.001 |
| 8 | M -> Y | Group parenting | Home environment | 0.731 | 0.140 | <.001 |
| 8 | M -> Y | Group parenting | Spanking | -0.042 | 0.095 | 0.662 |
| 8 | M -> Y | Group parenting | Not working | -0.064 | 0.088 | 0.468 |
| 9 | X -> M | Program | Education/training services | 0.216 | 0.021 | <.001 |
| 9 | M -> Y | Education/training services | Home environment | 0.129 | 0.032 | <.001 |
| 9 | M -> Y | Education/training services | Spanking | -0.055 | 0.024 | 0.024 |
| 9 | M -> Y | Education/training services | Not working | -0.185 | 0.022 | <.001 |
| 10 | X -> M | Program | Employment services | 0.184 | 0.019 | <.001 |
| 10 | M -> Y | Employment services | Home environment | 0.014 | 0.033 | 0.663 |
| 10 | M -> Y | Employment services | Spanking | -0.024 | 0.027 | 0.379 |
| 10 | M -> Y | Employment services | Not working | -0.03 | 0.025 | 0.241 |

Supplementary Table 3. Proportion of missing data for study variables, by EHS status

|  | **Controls** | | **EHS** | |
| --- | --- | --- | --- | --- |
| **Variable** | **Count** | **Percent** | **Count** | **Percent** |
| **Mediators** |  |  |  |  |
| Receipt of Employment services | 470 | 31.9% | 432 | 28.7% |
| Receipt of education/training services | 463 | 31.4% | 427 | 28.4% |
| Home visits > 1 times per month | 479 | 32.5% | 464 | 30.9% |
| Case management > 1 times per month | 474 | 32.2% | 442 | 29.4% |
| Any group parenting activity | 465 | 31.5% | 431 | 28.7% |
| Receipt of parenting education services | 515 | 34.9% | 434 | 28.9% |
| Receipt of parenting info in home visiting | 466 | 31.6% | 429 | 28.5% |
| Discussed parenting with case manager | 466 | 31.6% | 429 | 28.5% |
| Receipt of child development services in home visiting | 463 | 31.4% | 430 | 28.6% |
| **Outcomes** |  |  |  |  |
| Child Engagement during play | 690 | 46.8% | 628 | 41.8% |
| Child Attention during play | 692 | 46.9% | 629 | 41.8% |
| PPVT | 802 | 54.4% | 751 | 50.0% |
| Bailey MDI | 695 | 47.2% | 624 | 41.5% |
| Home total score | 606 | 41.1% | 564 | 37.5% |
| Spanking | 510 | 34.6% | 438 | 29.1% |
| Parent supportiveness during | 690 | 46.8% | 629 | 41.8% |
| Not employed or in school | 559 | 37.9% | 503 | 33.5% |

Supplementary Table 4*.* Descriptive statistics for EHSREP impact study covariates for participants with information on mediators and outcomes, by EHS status

| **Variable** | **Controls**  **(n=878)** | **EHS**  **(n=973)** | **P-value** |
| --- | --- | --- | --- |
| **Mediators** |  |  |  |
| Age at randomisation | 3.47 (4.60) | 3.62 (4.75) | 0.45 |
| Focus child is male | 443 (50.5%) | 497 (51.1%) | 0.79 |
| Focus child< 2500 grams | 42 (7.5%) | 58 (8.7%) | 0.45 |
| Race |  |  | 0.47 |
| White | 345 (40.1%) | 363 (37.9%) |  |
| Black | 281 (32.7%) | 328 (34.2%) |  |
| Hispanic | 192 (22.3%) | 230 (24.0%) |  |
| Other | 42 (4.9%) | 37 (3.9%) |  |
| Evaluated for concerns w/development | 37 (6.3%) | 37 (5.4%) | 0.49 |
| Age of mother at randomisation | 22.87 (5.78) | 22.82 (5.81) | 0.84 |
| 33% of federal poverty level | 195 (22.2%) | 237 (24.4%) | 0.28 |
| Previously in Head Start or Child dev program | 120 (14.3%) | 112 (12.0%) | 0.16 |
| Education level |  |  | 0.99 |
| Less than high school | 385 (45.6%) | 424 (45.3%) |  |
| HS or equivalent | 244 (28.9%) | 273 (29.1%) |  |
| More than HS | 215 (25.5%) | 240 (25.6%) |  |
| Moves in past year | 0.85 (1.12) | 0.85 (1.11) | 0.78 |
| Inadequate food | 56 (7.1%) | 40 (4.3%) | **0.010** |
| Inadequate housing | 97 (12.3%) | 110 (11.7%) | 0.70 |
| Inadequate money | 159 (20.2%) | 183 (19.8%) | 0.81 |
| Inadequate medical care | 110 (14.2%) | 117 (12.9%) | 0.46 |
| Received AFDC | 255 (30.7%) | 288 (30.9%) | 0.91 |
| Number of established biological or medical risks | 0.21 (0.68) | 0.18 (0.56) | 0.45 |
| Ever homeless in past year | 52 (6.8%) | 67 (7.3%) | 0.67 |
| Living arrangement |  |  | 0.29 |
| Lives with husband | 248 (28.4%) | 255 (26.2%) |  |
| Lives with other adults | 342 (39.2%) | 370 (38.0%) |  |
| Lives alone | 283 (32.4%) | 348 (35.8%) |  |
| Number of children in house | 0.47 (0.68) | 0.46 (0.72) | 0.48 |

.

Supplementary Table 5. Indirect effect estimates for the primary analysis and sensitivity analyses

|  |  | **Primary analysis** | | **Listwise deletion of missing data** | | **Adjustment for ‘inadequate food’** | |
| --- | --- | --- | --- | --- | --- | --- | --- |
| **Mediator** | **Outcome** | **Estimate** | **P-value** | **Estimate** | **P-value** | **Estimate** | **P-value** |
| Home visiting | Child eng | 0.063 | 0.029 | 0.057 | 0.087 | 0.072 | 0.015 |
| Home visiting | PPVT | 0.078 | 0.020 | 0.071 | 0.056 | 0.088 | 0.012 |
| Home visiting | MDI | 0.056 | 0.082 | 0.067 | 0.078 | 0.054 | 0.108 |
| Case management | Child eng | 0.087 | <.001 | 0.076 | 0.006 | 0.101 | <.001 |
| Case management | PPVT | 0.088 | 0.001 | 0.083 | 0.006 | 0.105 | <.001 |
| Case management | MDI | 0.065 | 0.012 | 0.042 | 0.175 | 0.075 | 0.008 |
| Group parenting | Child eng | 0.072 | 0.166 | 0.043 | 0.461 | 0.075 | 0.178 |
| Group parenting | PPVT | 0.091 | 0.003 | 0.094 | 0.151 | 0.228 | 0.001 |
| Group parenting | MDI | 0.103 | <.001 | 0.08 | 0.237 | 0.231 | <.001 |
| Education/training services | Child eng | 0.020 | 0.058 | 0.002 | 0.884 | 0.02 | 0.055 |
| Education/training services | PPVT | 0.014 | 0.232 | -0.001 | 0.942 | 0.018 | 0.134 |
| Education/training services | MDI | 0.030 | 0.012 | 0.013 | 0.325 | 0.029 | 0.017 |
| Employment services | Child eng | -0.001 | 0.939 | -0.011 | 0.303 | 0.006 | 0.586 |
| Employment services | PPVT | 0.000 | 0.965 | -0.006 | 0.596 | 0.009 | 0.447 |
| Employment services | MDI | 0.006 | 0.580 | -0.01 | 0.446 | 0.014 | 0.260 |
| Home visiting | Home environ | 0.072 | <.001 | 0.099 | <.001 | 0.074 | <.001 |
| Home visiting | Spanking | -0.036 | 0.013 | -0.045 | 0.024 | -0.039 | 0.009 |
| Home visiting | Not working | 0.012 | 0.386 | 0.017 | 0.354 | 0.01 | 0.459 |
| Case management | Home environ | 0.055 | 0.001 | 0.095 | <.001 | 0.067 | <.001 |
| Case management | Spanking | -0.040 | <.001 | -0.052 | <.001 | -0.043 | <.001 |
| Case management | Not working | 0.001 | 0.934 | -0.008 | 0.521 | -0.005 | 0.686 |
| Group parenting | Home environ | 0.103 | <.001 | 0.246 | <.001 | 0.207 | <.001 |
| Group parenting | Spanking | -0.012 | 0.663 | -0.014 | 0.639 | -0.012 | 0.679 |
| Group parenting | Not working | -0.018 | 0.468 | -0.032 | 0.255 | -0.04 | 0.145 |
| Education/training services | Home environ | 0.028 | <.001 | 0.029 | 0.001 | 0.029 | <.001 |
| Education/training services | Spanking | -0.012 | 0.028 | -0.011 | 0.081 | -0.013 | 0.016 |
| Education/training services | Not working | -0.040 | <.001 | -0.041 | <.001 | -0.038 | <.001 |
| Employment services | Home environ | 0.003 | 0.664 | 0.009 | 0.277 | 0.006 | 0.339 |
| Employment services | Spanking | -0.004 | 0.382 | -0.009 | 0.136 | -0.004 | 0.449 |
| Employment services | Not working | -0.005 | 0.245 | -0.01 | 0.097 | -0.007 | 0.193 |
